# Alteration of the cortical morphology in classical trigeminal neuralgia: voxel-, deformation-, and surface-based analysis

**DOI:** 10.1186/s10194-023-01544-x

**Published:** 2023-02-21

**Authors:** Xiuhong Ge, Luoyu Wang, Lei Pan, Haiqi Ye, Xiaofen Zhu, Sandra Fan, Qi Feng, Quan Du, Wenhua Yu, Zhongxiang Ding

**Affiliations:** 1 Department of Radiology, Affiliated Hangzhou First People’s Hospital, Zhejiang University School of Medicine, Hangzhou, 310000 People’s Republic of China; 2 Department of Radiology, Key Laboratory of Clinical Cancer Pharmacology and Toxicology Research of Zhejiang Province, Cancer Center, Affiliated Hangzhou First People’s HospitalZhejiang University School of MedicineShangcheng District, No.261, Huansha RoadZhejiang Province, Hangzhou, 310006 China; 3 Zhejiang Chinese Medical University, Hangzhou, China; 4 Department of Neurosurgery, Affiliated Hangzhou First People’s Hospital, Zhejiang University School of Medicine, Hangzhou, 310000 People’s Republic of China

**Keywords:** Classical trigeminal neuralgia, Cortex shape, Voxel-based morphometry, Deformation-based morphometry, Gray matter volume, Surface-based morphometry

## Abstract

**Objective:**

This study aimed to combine voxel-based morphometry, deformation-based morphometry, and surface-based morphometry to analyze gray matter volume and cortex shape in classical trigeminal neuralgia patients.

**Methods:**

This study included 79 classical trigeminal neuralgia patients and age- and sex-matched 81 healthy controls. The aforementioned three methods were used to analyze brain structure in classical trigeminal neuralgia patients. Spearman correlation analysis was used to analyze the correlation of brain structure with the trigeminal nerve and clinical parameters.

**Results:**

The bilateral trigeminal nerve was atrophied, and the ipsilateral trigeminal nerve volume was smaller than the contralateral volume in the classical trigeminal neuralgia. The gray matter volume of Temporal_Pole_Sup_R and Precentral_R was found to be decreased using voxel-based morphometry. The gray matter volume of Temporal_Pole_Sup_R had a positive correlation with disease duration and a negative correlation with the cross-section area of the compression point and the quality-of-life score in trigeminal neuralgia. The gray matter volume of Precentral_R was negatively correlated with the ipsilateral volume of the trigeminal nerve cisternal segment, cross-section area of compression point, and visual analogue scale. The gray matter volume of Temporal_Pole_Sup_L was found to be increased using deformation-based morphometry and had a negative correlation with the self-rating anxiety scale. The gyrification of the middle temporal gyrus_L increased and the Postcentral_L thickness decreased, as detected using surface-based morphometry.

**Conclusions:**

The gray matter volume and cortical morphology of pain-related brain regions were correlated with clinical and trigeminal nerve parameters. voxel-based morphometry, deformation-based morphometry, and surface-based morphometry complemented each other in analyzing the brain structures of patients with classical trigeminal neuralgia and provided a basis for studying the pathophysiology of classical trigeminal neuralgia.

**Graphical Abstract:**

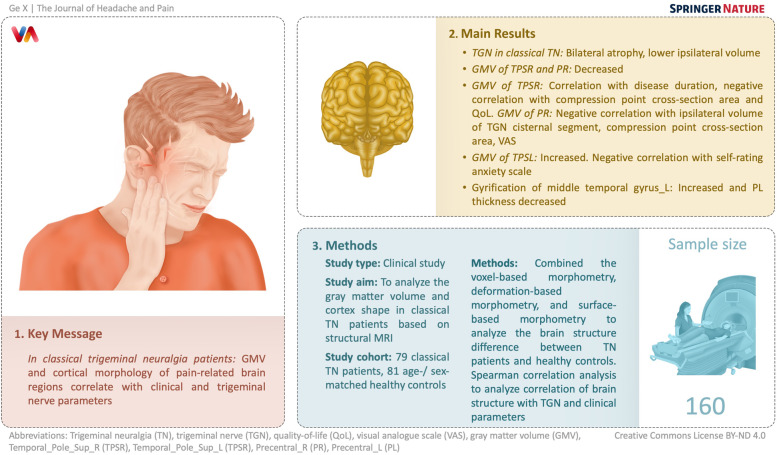

## Highlights

1. We first applied the three methods (VBM, DBM, and SBM) to the brain structure study in CTN and found that the three methods were complementary.
2. Some of the different brain regions correlated with clinical parameters, TGNcV, and CSA of the compression point.

## Introduction

Classical trigeminal neuralgia (CTN) is a chronic neurogenic pain distributed in the trigeminal sensory area [1–3], characterized by sudden, transient, electric shock-like pain [4]. Most patients present with purely paroxysmal pain, whereas some of them present with concomitant continuous pain [5, 6] which up to 49 percent [7, 8]. The pain is often triggered by the harmless actions in daily life (washing face, eating, brushing teeth, etc.) and is called the worst pain that human beings can endure [4].

According to the third edition of the International Classification of Headache Disorders (ICHD-3), CTN develops without any apparent cause other than neurovascular compression (NVC), which producing major morphological changes on the trigeminal nerve root [6, 9, 10]. NVC can cause demyelination of the nerve near the compression point, resulting in a short circuit between the fibers involved in pain production and those mediating light touch, causing the pain [11]. NVC can cause a reduction in trigeminal nerve (TGN) volume. A study of the postoperative histopathological parameters showed axon atrophy and demyelination of the TGN in patients with trigeminal neuralgia (TN) [12]. The imaging also showed that the ipsilateral TGN atrophy [1, 13–15] and the cross-sectional area (CSA) of the compression point were smaller [16] in patients with CTN compared with healthy controls (HCs).

A certain correlation existed between the TGN morphology and the changing brain structure of patients with CTN [17, 18]. Many recent studies showed that the brain structure changed in patients with CTN, which included the gray matter volume (GMV) [2, 12, 16, 19–27] and cortical morphology. The brain regions in which the GMV changed were mainly the frontal lobe, temporal lobe, parietal lobe, thalamus, hippocampus, and cerebellum; the GMV changed differently in different studies. The changes in cortical morphology mainly included the cortical thickness [19, 26, 28–32], local gyrification index (LGI) [28, 30], sulcal depth [30], surface area, and myelin level [31].

Voxel-based morphometry (VBM) compares the local GMV at the voxel level, converting brain images into standard space to compensate the global differences and preserve the local differences in the distribution of gray matter (GM) cortex [33, 34]. Deformation-based morphometry (DBM) characterizes the differences in the vector fields, which describe global differences in the brain shape [35]. The two methods provide a comprehensive assessment of anatomical differences throughout the brain cortex [36, 37]. DBM is more sensitive to the atrophy of subcortical areas [38, 39] and the anatomical information exists in the deformation field [40]. Some studies suggested that DBM could replace VBM [41]. Other studies also found that the brain regions detected by the two methods were similar; only the clusters were different [42].

Surface-based morphometry (SBM) matches the gyration and groove geometry to the spherical map of the expansion, which greatly reduces the potential misalignment caused by complex folding patterns and the change in global volume [43]. Different from VBM and DBM that analyze the GMV, SBM is used to examine different characteristics of the cortex, for example, cortical thickness, sulcal depth, surface area, gyrification, and so on [43]. The aforementioned three methods provided the measurement of the brain structure, not redundant information [43, 44]. They revealed different aspects of the brain structure, implying that combined the three methods may further contribution to the study of neurogenic diseases. The combination of the three methods focuses on the Parkinson's disease [42, 45], musical training [46], and chronic stroke [47], but has not been applied to CTN.

In this study, we combined VBM, DBM, and SBM to analyze the brain structure in patients with CTN. Our hypotheses were as follows. First, the GMV and cortical morphology of the pain-related brain regions in patients with CTN changed. Second, some of the changes in the brain included corrections in clinical parameters, such as visual analogue scale (VAS) scores, disease duration, and so on. Finally, the VBM, DBM, and SBM methods complemented each other in analyzing the brain structure of patients with CTN and providing a basis for studying CTN pathophysiology.

## Materials and methods

The local ethics committee of the Affiliated Hangzhou First People’s Hospital, Zhejiang University School of Medicine approved this prospective study (IRB# No. 202107002). The study was carried out following the Declaration of Helsinki. All the participants provided written informed consent.

### Participants

A total of 88 patients with CTN and 85 age- and sex-matched HCs were recruited from the Affiliated Hangzhou First People’s Hospital, Zhejiang University School of Medicine. The inclusion criteria for patients with CTN were as follows: (1) patients diagnosed with CTN according to ICHD-3 [6], which is that the CTN developing without apparent cause other than neurovascular compression [demonstration on MRI or during surgery of neurovascular compression (not simply contact), with morphological changes in the trigeminal nerve root]; (2) unilateral pain in the distribution of one or more branches of the TGN; (3) conventional magnetic resonance imaging (MRI) T1WI and T2WI sequence examinations revealing no evident abnormal brain signals; (4) no additional neurological or sensory deficits in all patients; (5) no previous surgical or other invasive procedures for CTN; (6) no contraindications to MRI; (7) aged 20–70 years; and (8) right-handedness. The exclusion criteria were as follows: (1) patients with CTN undergoing surgical treatment; (2) headaches, or other paroxysmal or chronic pain conditions; (3) a family history of headache or other types of pain in first-degree relatives; (4) other somatic or psychiatric conditions; and (5) contraindications to MRI. The inclusion criteria for the HCs were as follows: (1) age between 20 and 70 years; (2) good physical condition, with no history of tumors and mental diseases; (3) right-handedness; and (4) consent to join the study. The exclusion criteria were as follows: (1) headache or other chronic pain diseases; (2) MRI for contraindications, such as claustrophobia; and (3) severe hypertension, diabetes, and other diseases affecting brain function.

### Neuropsychological assessments

The psychiatrist evaluated the clinical psychological status of all participants. The quality-of-life score of patients with TN (TN QOLS) was used to assess the quality of life, including four dimensions: symptoms, physical function field, psychology (or emotion) field, and society (including family relationships) [48]. The mini-mental state examination (MMSE), the self-rating depression scale (SDS), and the self-rating anxiety scale (SAS) were used to evaluate cognitive function, depression, and anxiety symptoms.

### Pain evaluation

The VAS was used to assess the intensity of trigeminal neuralgia in patients with CTN in the last week. The researchers guided patients with CTN in rating their pain on a scale of 0–10 using a 10-cm ruler, with the higher score indicating greater pain intensity. A score of “0” represented no pain, and a score of “10” meant intolerable pain.

### MRI acquisition and analysis

All participants underwent MRI using a 3.0 T MRI scanner (Siemens, MAGNETOM Verio, Germany) and an eight-channel phased-array head coil. The 3D damage-gradient echo sequences were used to collect functional data. The parameters were as follows: $176\ structural\ images,\ repetition\ time\ (TR)=1900\ msec,\ echo\ time\ (TE)=2.52\ msec,\ thickness=1\ mm,\ field\ of\ view\ (FOV)=256\times256\ mm^2,\ voxel\ size=1\times1\times1\ mm^3,\ and\ turning\ angle=9\ degrees$. Trigeminal 3D volume interpolation body part examination (3D-VIBE) data were acquired using the following parameters: $TR=10\ ms,\ TE=3.69\ ms,\ flip\ angle=12^\circ,\ FOV=220\times220\ mm^2,\ voxel\ size=0.8\times0.8\times0.8\ mm^3,\ slice\ thickness=0.8\ mm,\ and\ 60\ slices$. The 3D short-time inversion recovery (3D-STIR) data were acquired using the following parameters: $SPC\ sequence,\ TR=3800\ ms,\ TE=194\ ms,\ FOV=230\times230\ mm^2,\ voxel\ size=0.9\times0.9\times0.9\ mm^3,\ slice\ thickness=0.9\ mm,\ and\ 64\ slices$.

#### Imaging processing

In this study, we used the three methods (VBM, DBM, and SBM) based on the computational anatomy toolbox 12.8.1 (CAT12.8.1, https://neuro-jena.github.io/cat/), an SPM12 extension with the default pipeline. For the 3D-T1WI data, we inspected each volume for any artifact that could affect the processing, such as segmentation, normalization, and so forth.

#### VBM analysis

The spatial adaptive nonlocal mean filter [49] was used as the first step because noise estimation and de-noising worked the best for original (non-interpolated) data. Then, the affine-registered (to further improve the outcomes of the segmentation) was made, and the center of mass was used to roughly correct for differences in the position between the image and the template. The 3D-T1WI were segmented into different tissue types, including GM, white matter (WM), and cerebrospinal fluid (CSF). Subsequently, we used the affine registration algorithm to record all the native-space tissue segments to the standard Montreal Neurological Institute (MNI) template and resample them to 1.5 × 1.5 × 1.5 mm^3^ [50, 51]. The use of the diffeomorphic anatomical registration through exponentiated algebra toolbox (DARTEL) was necessary to refine the inter-subject registration via the application of the diffeomorphic anatomical registration. The images were also modulated to preserve GM data and minimize the distortion of normalization. Finally, an 8-mm full width at half maximum (FWHM) Gaussian filter was applied to allow statistical analysis [19, 52, 53].

#### DBM analysis

The 3D-T1WI was converted into a DBM map for each participant using a procedure described previously [41]. The resulting nonlinear transformation from previous VBM analysis was inverted to obtain the deformation fields that mapped voxel coordinates in the subject native space (χ_1_, χ_2_, χ_3_) to equivalent voxels in the MNI template [*u*_1_(χ); *u*_2_(χ); *u*_3_(χ)]. Then, the Jacobian matrices of the deformation were generated and estimated using the first-order approximation. The Jacobian determinant minus one (|J| – 1) was calculated as the voxel-wise relative deformation value to create DBM maps. This value represented the factor by which each voxel of the participant’s brain expanded (positive value) or shrank (negative value) during registration to the MNI template [41] and was resampled to 1.5 × 1.5 × 1.5 mm^3^ [50, 51]. Finally, an 8-mm FWHM Gaussian filter was applied to allow statistical analysis.

#### SBM analysis

The analysis was semi-automated by applying default parameters for all processing steps, as described by Niddam et al. [43]. The cortical thickness was estimated using a projection-based methodology by calculating the distance between the inner (boundary between WM and GM) and outer (boundary between GM and CSF) cortical surfaces [19]. Importantly, this projection-based thickness allowed the appropriate handling of partial volume information, sulcal blurring, and sulcal asymmetries without explicit sulcus reconstruction [54]. This step provided the topological correction for defects in the surface mesh as well as for spherical inflation and spherical registration [30, 43]. The application of an adapted DARTEL algorithm during spherical registration enabled inter-subject analysis by mapping the matrix onto a standardized spherical surface. The right and left hemispheres were then merged into a single mesh, resampled to a template space (resolution 164 k mesh) [55], and spatially smoothed. Three additional geometric cortical measures, including sulcal depth (The sulcal depth refers to the depth of the grooves or "fissures" on the surface of the brain and is thought to reflect the folding pattern of the cerebral cortex), cortical complexity [Cortical fractal dimension is a measure of the complexity of the cerebral cortex, and it can be calculated by analyzing the pattern of folding on the surface of the brain and the cortical complexity (fractal dimension) was calculagted on the paper described in Yotter] [56], and gyrification (The gyrification is a measure of the complexity and folding of the cerebral cortex, which can be extracted based on the absolute mean curvature of the brain surface) [57], were also derived. A 15-mm FWHM Gaussian kernel was used for smoothing the cortical thickness images, while a 20-mm FWHM Gaussian kernel was used for other parameters as recommended [19, 43]. The smoothed images were used for between-group analyses, as described later.

#### TGN cisternal segment volume analysis

The 3D-VIBE or 3D-STIR images were used for the volume of the TGN cisternal segment (TGNcV) and CSA of the compression point analysis. The manual segmentation of the TGN cisternal segment from its emergence at the pons to its entry at Meckel’s cave slice by slice in the axial plane and CSA of the compression point (i.e., the entire cisternal segment) was performed with the uAI Research Portal (United Imaging Intelligence, China) embedded into the widely used package PyRadiomics (https://pyradio.mics.readthedocs.io/en/latest/index.html) by a junior physician with 4 years of experience and a senior physician with 9 years of experience. The TGNcVs were calculated for comparison in the following ways: ipsilateral and contralateral to the side of pain in patients with CTN; the average of HCs [(right + left)/2]. The analysis of delineation consistency between the junior and senior physicians was performed using the intraclass correlation coefficient (ICC).

Statistical analysis of clinical parameters and TGN structure.

The statistical analyses of clinical variables were conducted using SPSS software (version 26). The differences between groups were examined with independent-sample *t* tests for continuous data (age, VAS score, MMSE score, pain frequency, TGNcV, and so on) and with the chi-squared test for sex. The statistical significance was defined as *P* < 0.05.

#### Statistical analysis of structural images

The between-group differences in cortical thickness, sulcal depth, cortical complexity, gyrification (by SBM), and GMV (by DBM and VBM) were assessed with two-sample *t* tests controlling for age and sex. Total intracranial volume was also used as the covariate in the VBM models [49, 50, 58]. The two groups were compared in multiple ways: DBM and VBM based on GRF (voxels *P* < 0.001, clusters *P* < 0.05) and SBM based on family-wise error (FWE, voxels *P* < 0.001, clusters *P* < 0.05). Spearman correlations were performed on pain characteristics (disease duration, VAS, pain frequency, TN QOLS, SAS, SDS, etc.) and TGNcV for determining the correlations between mean cortical thickness/volume, gyrification from the clusters derived from VBM or DBM or SBM, and clinical parameters. The statistical significance was defined as *P* < 0.05, and all tests were two-tailed.

## Results

### Demographic information and clinical characteristics

**Fig. 1:**
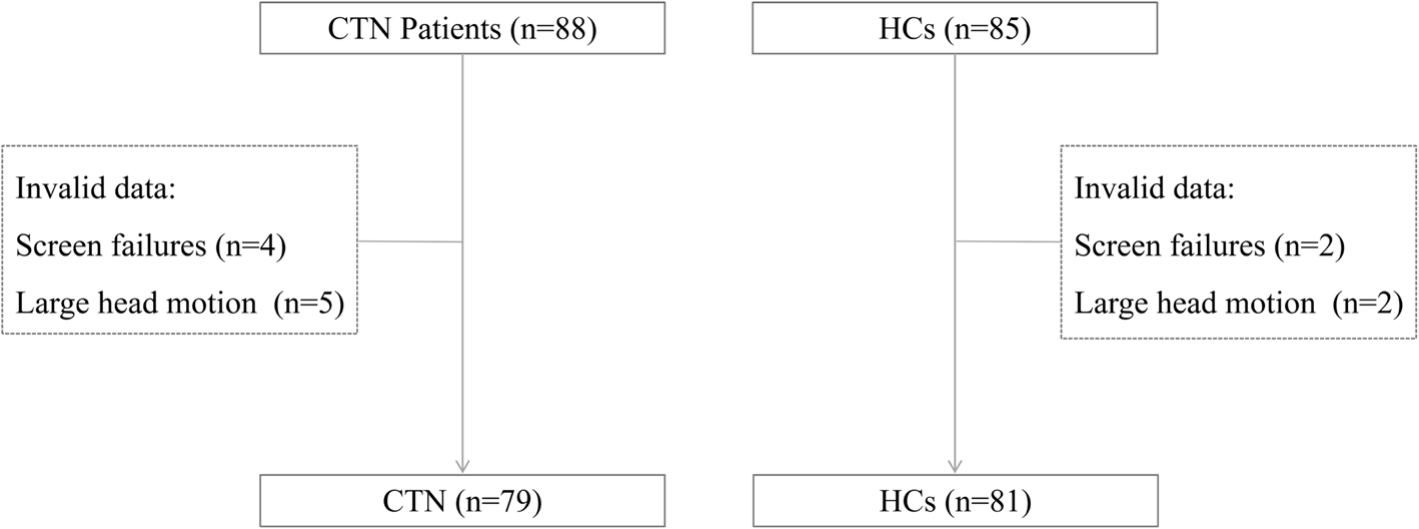
Selecion of patients with CTN and HCs. CTN, classical trigeminal neuralgia; HCs, healthy controls

The demographic variables and clinical characteristics of the participants are summarized in Table 1. A total of 79 patients with CTN (54F, 25 M; 54.05 ± 10.56 years old) and sex- and gender-matched 81 HCs (56F, 27F; 52.28 ± 8.56 years old) were included in this study. The inclusion process is shown in Fig. 1. All the patients with CTN had unilateral onset (52R, 27L), and the pain distribution was more in V2.3 (41/79). Most patients had severe pain intensity (VAS, 8.25 ± 1.77), and the average duration of attack was more than 2 min in 28 patients, and the peripheral or central sensitization may account for the continuous pain. Compared with HCs, patients with CTN had poorer quality of life and cognition and more severe depression and anxiety.

**Table 1:** Demographic and clinical characteristics of patients with CTN and healthy controls

	CTN		HCs	*P*
Sex (women/men)	54/25		56/27	0.957
Age (year)	54.05 ± 10.56		52.28 ± 8.56	0.077
Lateral (R/L)	52/27		NA	NA
Distribution	V2.3	41	NA	NA
	V3	15		
	V2	13		
	V1.2	8		
	V1.2.3	1		
	V1	1		
Average duration of attack	< 2 min	51	NA	NA
	> 2 min	28		
Attack frequency (per day)	< 20	24	NA	NA
	20–50	15		
	50–100	8		
	> 100	32		
Duration (year)	5.03 ± 4.94		NA	NA
Pain intensity (VAS)	8.25 ± 1.77		NA	NA
MMSE	26.94 ± 3.20		28.54 ± 1.82	0.000
Symptoms^*^	21.86 ± 4.36		6.00 ± 0.00	0.000
Physical function^*^	19.14 ± 4.27		13.95 ± 2.36	0.000
Psychology^*^	13.00 ± 4.22		5.67 ± 1.26	0.000
Society^*^	14.29 ± 4.09		5.00 ± 0.00	0.000
Self-rating depression scale	37.38 ± 7.11		28.59 ± 5.17	0.000
Self-rating anxiety scale	33.10 ± 5.91		28.37 ± 5.35	0.000
CSA of compression point (mm^2^)	11.18 ± 4.47		NA	NA
TGNcV (mm^3^)	Ipsi (53.90 ± 25.75)	Contra (68.17 ± 30.13)		0.002
	Ipsi (53.90 ± 25.75)		HCs (115.47 ± 31.59)	0.000
		Contra (68.17 ± 30.13)	HCs (115.47 ± 31.59)	0.000

*CTN* Classical trigeminal neuralgia, *HCs* Healthy controls; ^*^ the four dimensions of the quality-of-life score of patients with trigeminal neuralgia; *R* Right, *L* Left, *MMSE* Mini-mental state examination, *VAS* Visual analogue scale, *CSA* Cross-sectional area, *TGNcV* Volume of trigeminal nerve cisternal segment, *ipsi* ipsilateral, *contra* contralateral

### NVC degree and TGNcV

The ICC analysis showed that the TGNcV was in good agreement (ICC ≥ 0.75). All the patients displayed varying degrees of NVC on the side affected. The nerve distortion and/or displacement occurred in 5 patients and the significant indentation was found in 74 patients, which was caused by compression of the offending vessel.

**Fig. 2:**
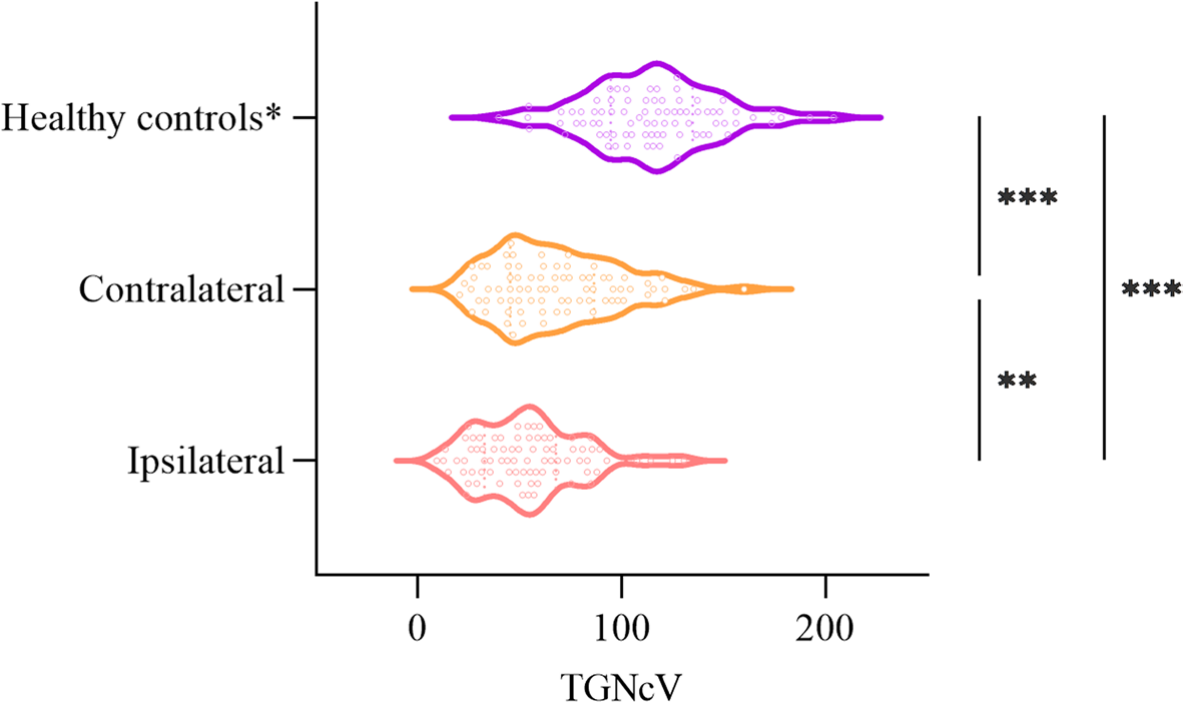
The TGNcV (mm^3^) between patients with CTN (ipsilateral and contralateral) and healthy controls. The ipsilateral TGNcV was significantly smaller compared with the contralateral TGNcV,and the bilateral TGNcVs were both significantly smaller compared with the average TGNcV of healthy controls. ^*^Average volume of the healthy controls ((right + left)/2); CTN, classical trigeminal neuralgia; TGNcV, volume of trigeminal nerve cisternal segment

Compared with the contralateral TGNcV, the ipsilateral TGNcV in patients with CTN was significantly smaller (53.90 ± 25.75 mm^3^ vs 68.17 ± 30.13 mm^3^, *P* = 0.002). Compared with the average TGNcV of the HCs, the nerves of the affected (115.47 ± 31.59 mm^3^ vs 53.90 ± 25.75 mm^3^, *P* = 0.002) or unaffected side (115.47 ± 31.59 mm^3^ vs 68.17 ± 30.13 mm^3^, *P* = 0.002) were all atrophied. The CSA of the compression point was 11.18 ± 4.47 mm^2^ (Table 1 and Fig. 2).

### VBM analysis between CTN and HCs

**Fig. 3:**
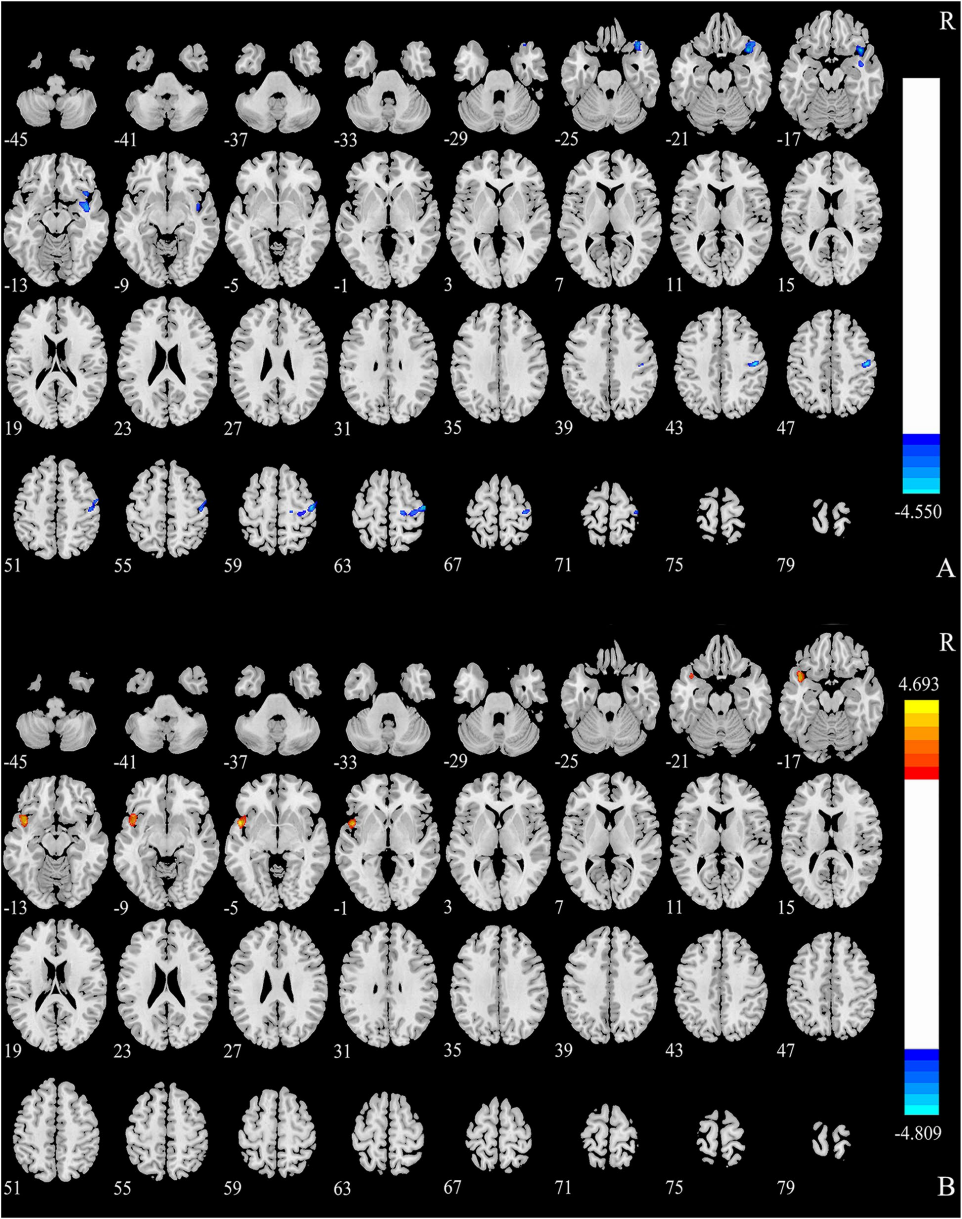
VBM and DBM analysis of the GMV. (A) VBM analysis revealed significantly decreased GMV in two clusters in patients with CTN compared with healthy controls. GMV atrophy was observed in the Temporal_Pole_Sup_R and Precentral_R (GFR, voxels *P* < 0.001, clusters *P* < 0.05). (B) DBM analysis revealed significantly increased GMV cluster (Temporal_Pole_Sup_L) in patients with CTN compared with healthy controls (GFR, voxels *P* < 0.001, clusters *P* < 0.05). CTN, classical trigeminal neuralgia; VBM, voxel-based morphometry; DBM, deformation-based morphometry; GMV: gray matter volume; GRF: Gaussian random field

**Fig. 4:**
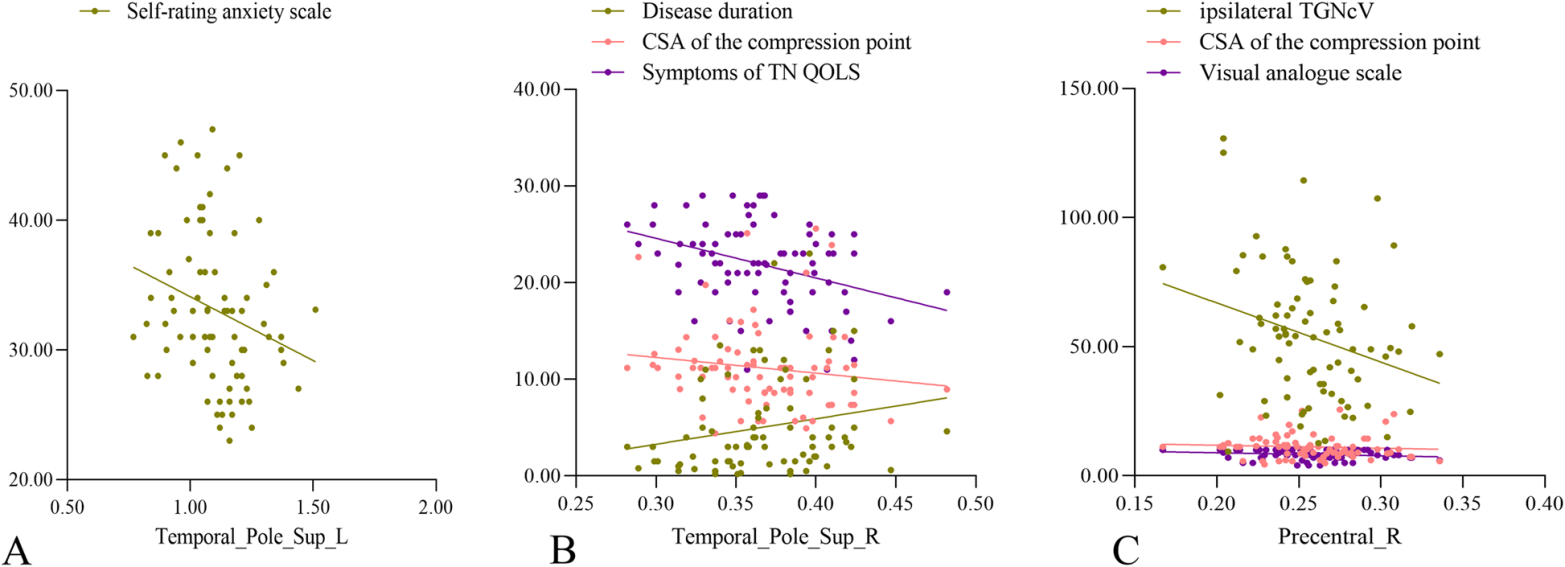
Correlations between the clinical parameters and brain regions in which the GMV changed in patients with CTN. (**A**) The GMV of TPOsup_L was negatively correlated with self-rating anxiety scale (*P* = 0.005, *r* = –0.341), (**B**) The GMV of TPOsup_R was positively correlated with disease duration (years) (*P* = 0.030, *r* = 0.245) and negatively correlated with the CSA of compression point (*P* = 0.048, *r* = –0.224) and symptoms of TN QOLS (*P* = 0.002, *r* = –0.336). (**C**) The GMV of precentral_R was negatively correlated with the ipsilatreal TGNcV (*P* = 0.013, *r* = –0.278), CSA of the compression point (*P* = 0.041, *r* = –0.231), and VAS (*P* < 0.025, *r* = –0.252). GMV, gray matter volume; CTN, classical trigeminal neuralgia; CSA, cross-sectional area; TN QOLS, quality-of-life score of patients with trigeminal neuralgia; TGNcV, volume of trigeminal nerve cisternal segment

The GM atrophy in patients was compared with that in HCs in two clusters: Temporal_Pole_Sup_R (10.07%) and Precentral_R (12.28%) (Fig. 3), as detected by VBM. Table 2 lists the standard space coordinates, different brain regions, and voxels of these brain regions. A positive correlation was found between the GMV of Temporal_Pole_Sup R and disease duration (year) (*P* = 0.030, *r* = 0.245). A negative correlation was found between the GMV of Temporal_Pole_Sup_R and CSA of the compression point (*P* = 0.048, *r* = –0.224) and symptoms of TN QOLS (*P* = 0.002, *r* = –0.336). A negative correlation was found between the GMV of precentral_R and ipsilateral TGNcV (*P* = 0.013, *r* = –0.278), CSA (*P* = 0.041, *r* = –0.231), and VAS (*P* < 0.025, *r* = –0.252) (Fig. 4).

**Table 2:** Brain regions in which the GMV or cortex shape changed in patients compared with healthy controls, as detected using VBM, DBM, and SBM

	Brain region	Side	Peak MNI coordinates			Cluster size (voxels)	Peak intensity
			*X*	*Y*	*Z*		
VBM	TPOsup	R	39	16.5	–18	787	–4.5498
	Precentral	R	48	–16.5	45	940	–4.5072
DBM	TPOsup	L	–51	7.5	–3	762	4.6929
SBM							
Gyrification	Middle temporal gyrus	L	–39.5	–0.58	–52.1	1523	4.1882
Thickness	Postcentral	L	–38.9	13.9	13.9	1802	–3.9562

*GMV* Gray matter volume, *CTN* Classical trigeminal neuralgia, *MNI* Montreal Neurological Institute, *VBM* Voxel-based morphometry, *DBM* Deformation-based morphometry, *SBM* Surface-based morphometry, *TPOsup* Temporal_Pole_Sup

### DBM analysis between CTN and HCs

The GM volume increased in patients compared with HCs in one cluster named Temporal_Pole_Sup_L (8.16%), as detected by DBM (Fig. 3). Table 2 lists the standard space coordinates, different brain regions, and voxels of these brain regions. A negative correlation was found between the GMV of Temporal_Pole_Sup_L and SAS (*P* = 0.005, *r* = –0.341) (Fig. 4).

### SBM analysis between CTN and HCs

**Fig. 5:**
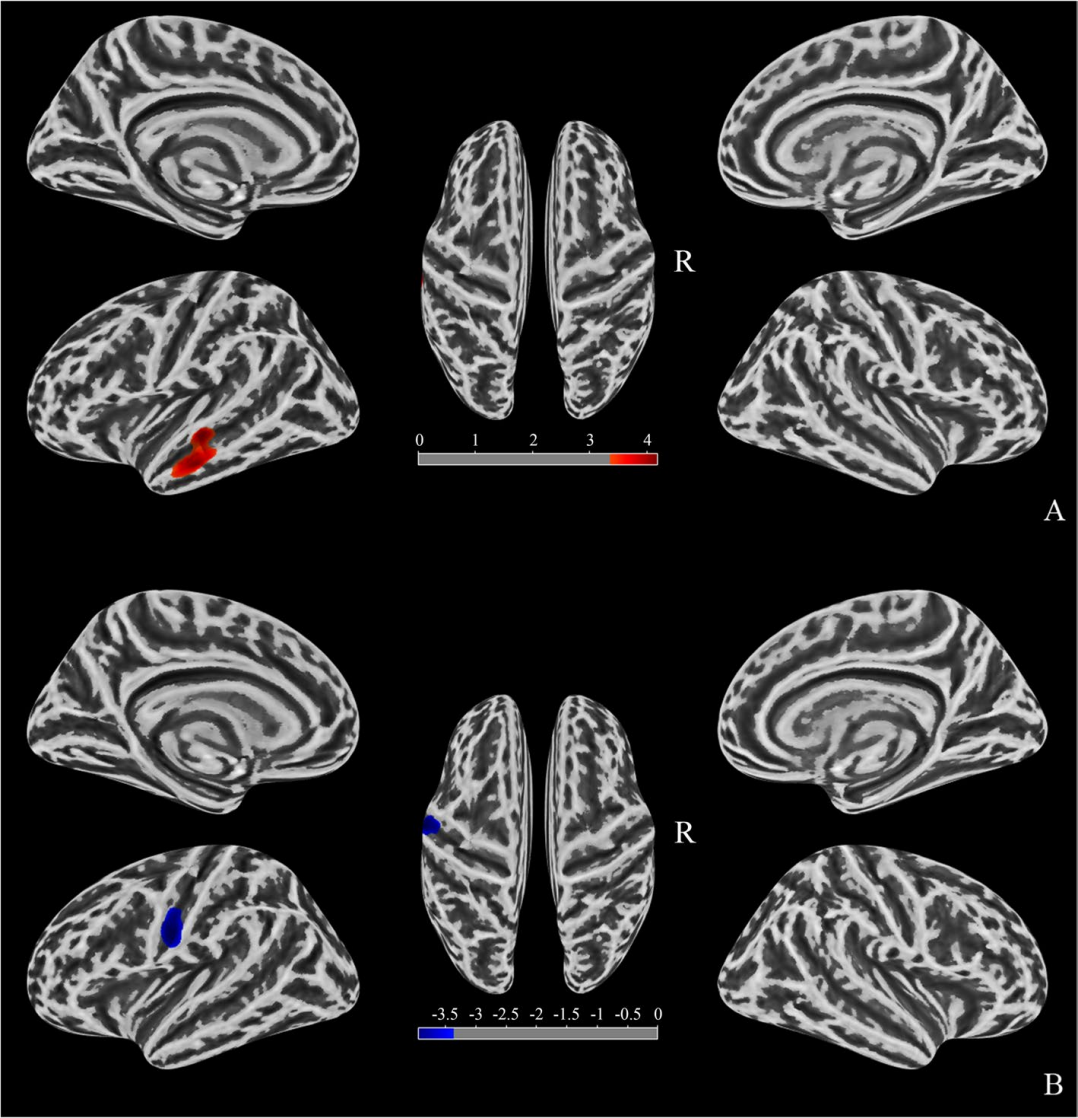
A comparison of the cortical morphology of patients with CTN and healthy controls using SBM. (**A**) Gyrification: red clusters representing significantly more gyrification (left middle temporal gyrus) in patients with CTN (FWE, voxels *P* < 0.001, clusters *P* < 0.05). (**B**) Cortical thickness: blue clusters representing significantly thinner cortical regions (left postcentral) in patients with CTN (FWE, voxels *P* < 0.001, clusters P < 0.05). CTN, classical trigeminal neuralgia; SBM, surface-based morphometry; FWE, family-wise error

The SBM analysis across the whole brain found that the gyrification increased in the Middle Temporal_L (3.70%) and the thickness of the cortex decreased in Postcentral_L (4.92%) of patients with CTN compared with HCs (Fig. 5).

## Discussion

This study involved a single-center, prospective assessment of 79 patients with CTN. The results showed that the brain regions detected by VBM, DBM, and SBM were different and complemented each other. Among these, some brain regions were correlated with the clinical parameters, TGNcV, and CSA of the compression point.

The three methods (VBM, DBM, and SBM) analyzed the change in brain neuroanatomy [42]. Among these, DBM and VBM were the voxel-level analysis methods [34, 59], which were used to measure the GMV [42]. VBM provided voxel-wise volume estimations of segmented GM, WM, and CSF [33, 37]. DBM relied on the deformation generated from the spatial registration to reflect the whole-brain structural changes [35, 60]. SBM analyzed the cortical morphological features based on vertex measurement and comparison of the cortical thickness [42]. These three brain structure analysis methods could extract different indicators, representing different structural characteristics of the brain cortex.

In this study, the GMV of Temporal_Pole_Sup_L was found to be decreased using VBM and increased using DBM. The Temporal_Pole_Sup plays an important role in pain memory, and the pain memory plays a crucial role in the perception of future pain [61, 62], indicating that the Temporal_Pole_Sup might be related to pain memory in patients with CTN. The GMV and side of Temporal_Pole_Sup were different between VBM and DBM due to two reasons. On the one hand, it might be some differences between the two methods. VBM converted images into standard space for information extraction, whereas the DBM method extracted information from deformation field. Therefore, it was seen that the two methods complemented each other. On the other hand, it might be the compensatory mechanism. CTN caused a decrease in the GMV of Temporal_Pole_Sup on the right side and an increase in the compensatory volume of Temporal_Pole_Sup on the left side. Pain is a subjective experience that can produce symptoms beyond the perception of pain itself [63]. Therefore, the reason for this phenomenon may also be caused by other factors, which needs further study. Wang et al. [17] found that the GMV of bilateral Temporal_Pole_Sup decreased. Li et al. [23] also found that the GMV of bilateral Temporal_Pole_Sup decreased and was negatively correlated with the course of disease. The previous studies on the GMV in patients with CTN mostly used VBM. Our study combined DBM and VBM to study the GMV in patients with CTN, which had a certain complementary effect on previous findings. The Temporal_Pole_Sup was also involved in emotion regulation and had neurocognitive functions [64]. The correlation analysis showed that the volume of Temporal_Pole_Sup_L decreased with the increase in anxiety. The CSA of the compression point and TN QOLS (symptoms dimension) decreased and the Temporal_Pole_Sup_R volume increased with the increase in disease duration. This indicated that the disease duration, CSA of compression point, TN QOLS (symptoms dimension), and mood might affect the Temporal_Pole_Sup GMV of patients with CTN.

In this study, besides the decrease in the GMV of Temporal_Pole_Sup _R, we also found a decrease in the GMV of precentral_R using VBM. The precentral_R is located in the frontal lobe and is the main motor area of the cerebral cortex. It is a key driver of motor output and associated with pain perception and regulation [12]. CTN is often triggered by harmless movements such as washing face and eating. Patients may limit the occurrence of such movements to avoid pain, which may lead to a change in precentral GMV. Thus, the precentral_R may reflect a sensory pain response caused by repeated CTN, motor inhibition of the maxilla, and facial muscle tone [65–67]. Tsai et al. [12] and Yan et al. [68] found that the GMV decreased in precentral_R. Wang et al. [20] found that the GMV decreased in multiple brain areas, including primary motor cortex and premotor area. The correlation analysis showed that the ipsilateral TGNcV and CSA of the compression point increased, and the pain degree of patients with CTN was more severe (VAS score was higher) with the decrease in the GMV of precentral_R. The indicated that the VAS score, ipsilateral TGNcV, and CSA of the compression point affected the GMV of precentral_R GMV.

In this study, we analyzed four parameters using SBM: sulcal depth, curvature, cortical thickness, and degree of gyrification. Among these, we found brain regions with different degree of gyrification and cortical thickness, while the remaining parameters displayed no changes. The middle temporal gyrus is the classic brain region of the default mode network. In this study, the gyrification of middle temporal_L increased. This was probably because the long-term pain stimulation led to a change in the morphology of the middle temporal gyrus (gyrification). Current studies on the brain structure of patients with TN only found a decrease in the GMV of the middle temporal gyrus. Li et al. [23] and Wang et al. [17] found that the GMV increased in the middle temporal gyrus, while Yan et al. [68] found that the GMV decreased in the middle temporal gyrus. No study showed a change in gyrification in the middle temporal gyrus, which might be caused by individual differences and different processing methods. The postcentral is the primary somatosensory cortex, which receives most somatosensory information from the thalamus and is involved in the anticipation, intensity, discrimination, spatial and temporal summation of pain processing, and pain coding [69]. In our study, we found a decrease in the cortical thickness of Postcentra_L. Desouza et al. [32] and Obermann et al. [21] found that the thickness of the left primary sensory cortex increased in patients with TN compared with HCs.

The ultrastructural features of neurons and other cells in the cortex may contribute to the morphological characteristics that are observed at a macroscopic level [70–72]. The cortical morphology characteristics, including GMV, cortical thickness, gyrification index, and sulcal depth have been indicated to reflect the regulation of intermediate progenitor cells’ genesis and amplification [30, 70]. The gray matter density, cortical area, channel pattern and cortical thickness affected the size of GMV [71]. Various cellular-level features including density, size, arrangement of neurons, nerve fibers, and neuroglia affected the cortical thickness [72]. Cortical gyrification adapts the cortical surface area to the skull, which promotes the development of neural circuits [73]. However, the exact relationship between ultrastructure and cortical morphological characteristics remains an active area of investigation, and more research is needed to fully understand this relationship. 

## Conclusions

In this study, we analyzed the brain structure of patients with CTN by combining VBM, DBM, and SBM. We found that some brain regions in these patients correlated with clinical parameters, TGNcV, and CSA of the compression point. However, no overlapping brain regions were found among the three methods. This might be because the processing procedures of the three methods were different, which also indicated that the three methods complemented each other. The analysis of CTN using the combination of the three methods could provide additional information and a basis for further investigation of the pathophysiology of CTN.

## Limitations

In this study, the features of the recruited patients, for example, disease duration, pain distribution, pain intensity and so on, were not consistent. However, we could not perform a subgroup analysis due to sample size limitations. The sample size should be further expanded for stratified group research in the future. The other limitation was that our study was a cross-sectional study and the longitudinal data were not included, although this was a general characteristic of most studies. Further studies should be combined with the longitudinal data to further clarify the pathophysiological mechanism of CTN.

## Abbreviations

CTN: Classical trigeminal neuralgia
ICHD-3: The third edition of the International Classification of Headache Disorders
NVC: Neurovascular compression
TGN: Trigeminal nerve
TN: Trigeminal neuralgia
CSA: Cross-sectional area
HCs: Healthy controls
GMV: Gray matter volume
LGI: Local gyrification index
VBM: Voxel-based morphometry
GM: Gray matter
DBM: Deformation-based morphometry
SBM: Surface-based morphometry
VAS: Visual analogue scale
MRI: Magnetic resonance imaging
TN QOLS: Quality-of-life score of patients with TN
MMSE: Mini-mental state examination
SDS: Self-rating depression scale
SAS: Te self-rating anxiety scale
TR: Repetition time
TE: Echo time
3D-VIBE: 3D volume interpolation body part examination
3D-STIR: 3D short-time inversion recovery
GRF: Gaussian random fields
WM: White matter
CSF: Cerebrospinal fluid
MNI: Montreal Neurological Institute
FWHM: Full width at half maximum
FWE: Family-wise error
TGNcV: The volume of the TGN cisternal segment
